# Novel Survivin Peptides Screened With Computer Algorithm Induce Cytotoxic T Lymphocytes With Higher Cytotoxic Efficiency to Cancer Cells

**DOI:** 10.3389/fmolb.2020.570003

**Published:** 2020-09-02

**Authors:** Qiuqiang Chen, Gang Jia, Xiaolei Zhao, Ying Bao, Yu Zhang, Cengiz Ozkan, Boris Minev, Wenxue Ma

**Affiliations:** ^1^Key Laboratory for Translational Medicine, The First Hospital Affiliated to Huzhou University School of Medicine, Huzhou, China; ^2^Department of Oncology, Henan Provincial People’s Hospital, People’s Hospital of Zhengzhou University, Zhengzhou, China; ^3^Department of Urology, Huaihe Hospital of Henan University, Kaifeng, China; ^4^Mechanical and Automotive Engineering, School of Engineering, RMIT University, Melbourne, VIC, Australia; ^5^Materials Science and Engineering Program, Department of Mechanical Engineering, University of California, Riverside, Riverside, CA, United States; ^6^Department of Medicine, Moores Cancer Center, University of California, San Diego, La Jolla, CA, United States

**Keywords:** survivin, peptide, cytotoxic T lymphocyte, polymeric nanoparticle, cancer immunotherapy

## Abstract

The identification of novel biomarkers and therapeutic targets in advanced cancer is critical for improving cancer diagnosis and therapeutics. Survivin (SV) is highly expressed predominantly in most cancer cells and tissues but is absent or undetectable in terminally differentiated normal adult tissues. Therefore, it functions as an almost universal tumor antigen. Peptides are short chains of amino acids linked by peptide bonds. To obtain novel SV decamers that are able to induce SV-specific cytotoxic T lymphocytes (CTLs) with a higher cytotoxic efficiency against cancer cells, major histocompatibility complex (MHC) peptide binding algorithms were conducted to predict nine modified SV_95_ decamers (from SV_95–2_ to SV_95–10_) based on the natural SV_95–104_ peptide sequence of ELTLGEFLKL (here defined as SV_95–1_). The fluorescent density of each SV_95_ peptide was determined by a MHC stability assay, followed by the generation of SV_95_-specific CTLs with each SV_95_ peptide (from SV_95–1_ to SV_95–10_) and human dendritic cells (DCs) loaded with Poly(lactic-*co*-glycolic) acid (PLGA) nanoparticles encapsulated with SV_95_ peptide. Finally, IFN-γ ELISpot and CytoTox 96^®^ Non-Radioactive Cytotoxicity Assays were employed to verify their cytotoxic efficiency of the SV_95_-specific CTLs generated with the corresponding artificial antigen presenting cells (aAPCs) containing SV_95_ (SV_95–1_ to SV_95–10_) peptide. Furthermore, the cytotoxicity of the SV_95_ specific CTLs generated with nine mutated SV_95_ peptides was compared to the one generated with natural SV_95–1_ peptide and TIL2080 *cells*. The results indicated that the HLA-A2-restricted mutated SV_95_ epitope decamers (SV_95–6_ and SV_95–7_) showed significant higher binding ability compared to natural peptide SV_95–1_ in MHC stability assay. More importantly, SV_95–_specific CTLs with higher cytotoxicity were successfully induced with both SV_95–6_ and SV_95–7_ peptides, which significantly eliminated target cells (not only SV_95–1_ peptide pulsed T2 cells, but also both HLA-A2 and SV positive cancer cells) when compared to those generated with natural SV_95–1_ peptide and TIL2080 cells. These findings suggest that the SV_95–6_ and SV_95–7_ peptides are two novel HLA-A2-restricted CTL epitopes and may be useful for the immunotherapy for patients with survivin expressing cancer.

## Introduction

Survivin (SV) is a protein encoded by baculoviral inhibitor of apoptosis repeat-containing 5 (BIRC5) gene in humans (Rahban et al., 2019). SV is the smallest member of the mammalian inhibitors of apoptosis protein (IAP) family of protein (Garg et al., 2016), and has the capability to inhibit caspase-3, caspase-7, and caspase-9 in cells; therefore, its overexpression can lead to resistance to cell death caused by various apoptotic stimuli (Shin et al., 2001). SV is also one of the most specific cancer antigens identified to date because it has been reported to be overexpressed in almost all cancer types with different expression levels (Rafatmanesh et al., 2020), including lung cancer (Marwitz et al., 2019), breast cancer (Gao et al., 2018), gastric cancer (Liu et al., 2019), bladder cancer (Zhang et al., 2019), prostate cancer (Hwang et al., 2020), melanoma (Fenstermaker et al., 2018), colorectal cancer (Or et al., 2020), hepatocellular carcinoma (Nasr et al., 2019), and malignant lymphoma (Miletic et al., 2016). However, it is absent or undetectable in terminally differentiated normal adult tissues (Hirohashi et al., 2002) with the exception of activated T lymphocytes (Leung et al., 2007), erythroblasts (Keerthivasan et al., 2012), and self-renewing stem cells (Martini et al., 2016). Expression of SV in cancers correlates with not only inhibition of apoptosis and a decreased rate of cell death, but also resistance to chemotherapy and aggressiveness of cancers (Garg et al., 2016). As a result, SV remains a promising target and biomarker for drug discovery and cancer therapeutics. As a matter of fact, the most advanced cancer therapeutic agents, antisense oligonucleotides and YM155 (a SV inhibitor) related to SV were halted after multiple clinical trials due to either low antitumor efficacy and/or over toxicity issues (Li et al., 2019b).

Cytotoxic T lymphocytes (CTLs) are a functional subgroup of the cellular arm of antigen-specific immune responses. Tumor antigen-specific CTLs play a critical role in eliminating cancer cells by not only inducing long-term tumor regression in a tumor-associated antigen (TAA) fashion, but also depending on a cell contact manner (Li et al., 2017; Wang et al., 2018; Chen et al., 2019). CD8^+^ CTLs eliminate malignant cells through recognition by T cell receptors (TCRs) of specific antigenic peptides presented on the surface of cancer cells by major histocompatibility complex class I (MHC I)/β-2-microglobulin complexes, and through killing of target cells mainly by releasing the content of secretory lysosomes containing granule exocytosis (i.e., perforin) and granule-associated enzymes (i.e., granzyme B) (Durgeau et al., 2018), as well as using death ligands/death receptor system (Martinez-Lostao et al., 2015). Most CTLs express TCRs that can recognize specific antigens (peptides) often produced by cancer cells or viruses and capable of stimulating immune responses. CD8^+^ CTLs recognize these tumor antigens as small peptides bound to cell surface molecules encoded by the MHC I molecules (Pardoll, 2000). Therefore, peptides and their affinity to MHC I molecules are important factors for the generation of tumor antigen-specific CTLs (Wu et al., 2019).

T cell-based immunotherapy has been considered as a promising non-invasive treatment option for cancer that could be used to treat minimal residual disease, to prevent metastatic spread, or to delay recurrences without compromising quality of life. Although T cells genetically equipped with chimeric antigen receptors (CARs) or TCRs have shown remarkable effectiveness in treating some hematological malignancies, the efficacy of engineered T cells in treating solid tumors is still far from satisfactory (Li et al., 2019a). Nevertheless, TAA cytotoxic T cells represent a new, potentially effective and non-toxic therapeutic approach for patients with relapsed or refractory solid tumors (Hont et al., 2019).

In most cases, CTL epitopes are naturally processed peptides bound and presented by MHC molecules. Thus, antigenic peptides and their affinity for binding to MHC molecules are equally crucial for the production of tumor antigen specific CTLs. Natural SV peptides including SV_95–104_ (defined as SV_95–1_ in this study, ELTLGEFLKL) and SV 2B80-88 (AYACNTSTL) have been reported to successfully induce SV-specific CTLs not only in research laboratories (Turksma et al., 2013; Li and Ding, 2015; Shionoya et al., 2017) but also clinically (Tsuruma et al., 2004; Tanaka et al., 2013; Shima et al., 2018). Although SV-targeted dendritic cell (DC)-based cancer immunotherapy is a promising clinical strategy, SV-specific CTLs immunotherapy still faces many challenges in clinical application such as low therapeutic efficacy (Li and Ding, 2015).

To improve therapeutic and potent anti-tumor efficacy of tumor antigen specific CTLs, we used human DCs and SV_95_ peptide loaded poly (lactic-*co*-glycolic) acid nanoparticles (PLGA-NPs) to make artificial antigen-presenting cells (aAPCs) and have demonstrated that aAPCs are superior to peptide pulsed DCs when inducing CTL lines due to the aAPCs’ capability to continuously present tumor antigens to T cells in a sustained fashion (Li et al., 2017). Because knowledge of the peptides presented on MHC I molecules is fundamental for the development of highly specific T cell-based immunotherapies, we have attempted to further search for novel SV peptide sequences with stronger immunogenicity by artificially changing the peptide sequences based on natural SV_95–1_ sequence. Furthermore, the epitope peptides are typically composed of 8 to 10 amino acids, 2 to 3 main anchor residues interact with MHC class I molecules and 2 to 3 amino acid residues bind to TCRs (Wieczorek et al., 2017). With the aid of computer algorithms and based on natural SV_95–1_ peptide sequence, nine novel CTL epitopes that predict the ability of SV to specifically bind to the MHC class I allele were identified. To complete this study, T2 peptide binding assay was firstly performed to examine the binding stability of the SV_95_ peptides with MHC class I molecules. Next, SV_95_-specific CTLs were generated by using their corresponding SV_95_ peptides from SV_95–1_ to SV_95–10_ and aAPCs. Afterward, cytotoxicity of the nine novel CTL lines was examined and compared to the one generated with natural SV_95–1_ peptide, and TIL2080 cell line that was used as the systemic internal control.

## Materials and Methods

### Peptides, Cytokines and Antibodies

All the peptides identified as potential antigens, including HLA-A^∗^0201 restricted peptides of Mart-1_27–35_ (Mart-1, AAGIGILTV), HIV pol peptide (positive control peptide, ILKEPVHGV), natural SV_95–104_ (SV_95–1_, ELTLGEFLKL) and other nine algorithm designed novel SV_95_ peptides from SV_95–2_ to SV_95–10_ with the sequences summarized in Table 1, were synthesized at GenScript Corporation (Piscataway, NJ, United States). The purity (>95%) and identity of these peptides were determined by analysis of analytic HPLC and mass spectrometry. These peptides were dissolved in DMSO at a stock concentration of 20 mg/ml, aliquoted in 100 μl, and stored in −20°C freezer. Recombinant human granulocyte-macrophage colony-stimulating factor (GM-CSF) and recombinant human interleukins including IL-2, IL-4, and IL-7 were purchased from R&D Systems (Minneapolis, MN, United States). Monoclonal antibodies including CD3, CD8, CD45, CD56 were purchased from BD Biosciences (San Jose, CA, United States). CD28 (Clone CD28.2) was purchased from eBioscience (San Diego, CA, United States). Human anti-IFN-γ antibody was purchased from Mabtech AB (Nacka Strand, Sweden); anti-MHC class I (clone BB7.2) and anti-MHC class II (clone IVA12) antibodies were purchased from BD Pharmingen (San Diego, CA, United States).

**TABLE 1:** SV_95_ peptide sequences for evaluation.

Peptide ID	Designation	Sequence	Predicted half-life*
SV_95–1_	SV_95_ (95–104)	ELTLGEFLKL	3.044
SV_95–2_	SV_95_-10V	ELTLGEFLKV	9.911
SV_95–3_	SV_95_-2V10V	EVTLGEFLKV	0.867
SV_95–4_	SV_95_-3Y	ELYLGEFLKL	9.741
SV_95–5_	SV_95_-1Y3Y	YLYLGEFLKL	597.464
SV_95–6_	SV_95_-1X3X	XLXLGEFLKL	1549.671
SV_95–7_	SV_95_-1X3X10X	XLXLGEFLKX	1945.230
SV_95–8_	SV_95_-1Y3W10V	YLWLGEFLKV	5045.441
SV_95–9_	SV_95_-1Y2V3Y10V	YVYLGEFLKV	170.208
SV_95–10_	SV_95_-1Y2V3W10V	YVWLGEFLKV	441.476

*For patent purpose, X in this table stands for an amino acid.*

### Polymers and Fluorescent Dye

Poly(lactic-*co*-glycolic) acid (PLGA) is a copolymer that has been approved by the U.S. Food and Drug Administration (FDA) for use in therapeutic devices due to its biodegradability and biocompatibility. The PLGA used in this study was a copolymer with a composition ratio of 50% lactic acid and 50% glycolic acid, and a molecular mass of 23000. Polyvinyl alcohol (PVA), a water-soluble synthetic polymer used as an emulsifying agent (average MW 30000–70000), and lipopolysaccharide (LPS) were purchased from Sigma-Aldrich (St Louis, MO, United States). Coumarin 6, a fluorescent dye exhibits green light (500 nm) purchased from Polysciences Inc. (Warrington, PA, United States), was applied as an indicator in nano drug delivery systems.

### Human Cell Lines and Human Cells

T2 (ATCC, TAP^–^, CRL-1992^TM^) cell line, a B-lymphoblast, expresses neither HLA-DR nor major histocompatibility (MHC) class II antigen. However, its expression of HLA-A2 and CD7 is beneficial toward antigen-processing study and T-cell recognition of MHC class I antigens. Both the HLA-A2 and SV positive cancer cell lines used in this study included LNCap (ATCC, CRL-1740^TM^), which was derived from a metastatic prostate patient; A549 (ATCC, CCL-185^TM^), which was derived from a lung cancer patient; MCF7 (ATCC, HTB-22^TM^), which was from a breast cancer patient. The HLA-A2 negative and SV positive prostate cancer cell line used in this study was PC-3 (ATCC, CRL-1435^TM^), which was derived from a patient with metastatic prostate cancer. All of the aforementioned cancer cell lines were kept in RPMI 1640 medium supplemented with 10% fetal bovine serum (HyClone, Logan, UT, United States), 1% GlutaMAX and 1% Pen Strep (Life Technologies, Carlsbad, CA, United States). TIL2080, a tumor-infiltrating lymphocyte cell line recognizing Mart-1 was kindly provided by Dr. John Wunderlich (NIH/NCI, Bethesda, MD, United States) and was maintained in RPMI 1640 medium supplemented with 10% human AB serum (Omega Scientific, Tarzana, CA, United States), IL-2 (20 U/ml) and IL-7 (30 U/ml) (R&D, Minneapolis, MN, United States). PBMCs from five healthy donors were provided by San Diego Blood Bank and Scripps Green Hospital (San Diego, CA, United States). Normal human umbilical cord blood (UCB) CD34^+^ cells were purchased from AllCells (Alameda, CA, United States). This study was approved by Institutional Review Board at University of California San Diego.

### MHC I Stability Assay

*T2* peptide *binding assay* was performed as described (Zirlik et al., 2006) with a little modification, and was used to assess the binding ability of all the peptides including positive control peptide and 10 of the SV_95_ peptides. T2 cells (TAP^–^, HLA-A0201^+^) were cultured in complete RPMI medium. A number of 1 × 10^6^ cells were added into 1 ml AIM-V medium (Life Technologies, Carlsbad, CA, United States) and incubated with human β2-microglobulin (EMD Biosciences, San Diego, CA, United States) at 3 μg/ml and peptide including either positive control peptide (POL476, ILKEPVHGV) or each SV_95_ peptide (from SV_95–1_ to SV_95–10_) at various final concentrations of 10, 0.1, and 0.01 μM at 37°C for 21 h. One day later, T2 cells were incubated with anti-HLA-A2.1 (BB7.2) mAb at a saturating concentration for 30 min at 4°C. The samples were then washed twice with FACS buffer (2% fetal calf serum (FCS) in Hank’s Balanced Salt Solution with addition of 2 mM EDTA), fixed with 1% paraformaldehyde (PFA) diluted in HBSS. Finally, the samples were analyzed with a flow cytometer (Becton Dickinson, Immunocytometry systems, San Jose, CA, United States). The mean fluorescence intensity (MFI) observed for each peptide concentration (after division of the MFI observed without peptide) was used as an estimate for peptide binding and expressed as a fluorescence index.

### Preparation of Activated PBMCs

PBMCs were prepared at 1 × 10^6^/ml in complete RPMI medium. After washing the 96-well plate with sterile PBS, 100 μl of the PBMC cell suspension was added to each well. Soluble anti-CD28 mAb (Clone CD28.2, eBioscience) was then added into each well at a concentration of 2 μg/ml. The plate was incubated at 37°C and 5% CO_2_ for 2–3 days, then, the cells were harvested and processed for further experiments.

### Fabrication of PLGA Nanoparticles Encapsulated SV_95_ Peptide

As we previously described, a double emulsion-solvent evaporation technique was used to fabricate PLGA nanoparticles (PLGA-NPs) encapsulated SV_95_ peptide (Li et al., 2017). The particle size was analyzed using a Zetasizer^®^ Nano ZS90 (Malvern Instruments, Worcestershire, United Kingdom), and the zeta potential of the PLGA-NPs was measured using ZetaPlus^TM^ (Brookhaven Instruments Corporation, Holtsville, NY, United States). The peptide loading and peptide encapsulation efficiency parameters have been previously described (Li et al., 2017). The SV_95_ peptide encapsulated in each product of PLGA-NPs was one of the 10 SV_95_ peptides in Table 1. Each type of PLGA-NPs loaded with their corresponding SV_95_ peptide was used for making aAPCs.

### Generation of aAPCs

A detailed method for the generation of aAPCs had been described in our previous publications (Li et al., 2017). Briefly, human imDCs were generated from HLA-A2 positive PBMCs. Enriched monocytes were cultured in RPMI medium supplemented with 10% FCS, recombinant human GM-CSF at 1000 U/ml and recombinant human IL-4 at 400 U/ml (R&D, Minneapolis, MN, United States) at 37°C and 5% CO_2_. The same concentration of GM-CSF and IL-4 was added into the medium, respectively, on day 3. The imDCs were ready on day 5 in the aggregates of loose adherent cells. Human imDCs at a concentration of 50000 cells/ml were incubated with coumarin 6-labeled PLGA-NPs at 100 μg/ml in a 12-well plate for 45 min. After removing the uninternalized PLGA-NPs by washing with PBS, the imDCs were stained with Hoechst 33342 (Invitrogen, Carlsbad, CA, United States), followed by analysis with a confocal microscope (Leica TCS SP2, Buffalo Grove, IL, United States). To make the end-product of aAPCs, the above human imDCs were further incubated with PLGA-NPs encapsulated SV_95_ peptide (PLGA-NPs-SV_95_) for an hour, followed by maturation with the addition of lipopolysaccharides (LPS, Sigma-Aldrich, St. Louis, MO, United States) at 100 ng/ml for additional 2 days (Ma et al., 2012; Li et al., 2017). These cells were aAPCs, collected on day 7 for further evaluation (e.g., quality control examination), and used for the generation of SV_95_-specific CTLs.

### *In vitro* Generation of SV_95_-Specific CTLs

Human CD8^+^ cells were first isolated from HLA-A2 positive healthy donors using CD8 MicroBeads (Miltenyi Biotec, Auburn, CA, United States). The aAPCs were then added to CD8^+^ T cells at a ratio of 1 to 10 in RPMI 1640 medium supplemented with 10% human AB serum. IL-2 at the concentration of 20 U/ml and IL-7 at the concentration of 30 U/ml were added to the culture medium after day 3. On day 7, the first restimulation was carried out. The stimulator cells (autologous CD8^–^ cells) irradiated with Cesium (Cs) at the dose of 50 Gray (Gy) were mixed with effector cells (CD8^+^ T cells) with an addition of both β2-microglobulin and peptide at a concentration of 5 μg/ml, respectively. On day 10, half of the culture medium was replaced with fresh CTL media containing the same dose of IL-2 and IL-7. The second restimulation was repeated with the same protocol on day 14. Eventually, the SV_95_-specific CTLs were collected and examined on day 21 using both Enzyme-linked immunospot (ELISpot) and CytoTox 96^®^ Non-Radioactive Cytotoxicity assays (Ma et al., 2012; Li et al., 2017).

### ELISpot Assay

IFN-γ production was evaluated to reflect activity of the SV_95_-specific CTLs that release IFN-γ cytokine against target cells. Human IFN-γ ELISpot assay was performed according to the protocol, as we previously reported (Li et al., 2017; Shao et al., 2017). ELISpot plates (Millipore, Billerica, MA, United States) were coated with 10 μg/ml monoclonal human anti-IFN-γ antibody (Mabtech, Nacka Strand, Sweden) and incubated at 4°C overnight. The culture plates were washed with PBS on the second day and then blocked with the medium containing 10% human AB serum at 200 μl/well for 2 h at 37°C. T2 cells at a number of 2 × 10^4^ in 80 μl/well were pulsed with Mart-1 peptide (20 μl of 5 μg/ml), and TIL2080 cells at a number of 2 × 10^4^/well in 100 μl were added in, and used as a system positive control. T2 cells pulsed with SV_95–1_ peptide with the addition of SV_95–1_-CTLs were used as the internal positive control. To test the activity of SV_95_-specific CTLs generated from each SV_95_ peptide (SV_95–1_ to SV_95–10_), T2 cells pulsed with SV_95–1_ peptide were included as target cells. In addition, a pair of prostate cancer cell lines including PC-3 and LNCap were also used as target cells. LNCap cell line expresses both SV and HLA-A2 molecules while PC-3 cell line expresses only SV protein. According to our previous optimized condition, 1 × 10^5^–8 × 10^5^ cells/well provided a linear result. In order to obtain an expected spot counts, the target cell number, including Mart-1 or SV_95–1_ peptide pulsed T2 cells or cancer cells used in these experiments, was 2 × 10^4^/well, the effector cells including TIL2080 or SV_95_-specific CTLs were loaded at an optimized number of 2 × 10^4^/well (E:T = 1:1). All cultures were carried out in triplicate and the plates were incubated at 37°C for 20 h. The biotinylated secondary antibody for IFN-γ was added in each well after washing with PBS, and then incubated at 37°C for additional 2 h. The avidin-biotinylated horseradish peroxidase complex was added and incubated at room temperature for another 1 h. The substrate solution at 100 μl/well was added and incubated for 3 min, and the reaction was stopped with running tap water. IFN-γ spots on the plate membrane were counted with an ELISpot reader (CTL Analyzers LLC, Cleveland, OH, United States).

### Cytotoxicity Assay

As we previously described (Ma et al., 2011, 2012; Li et al., 2017; Chen et al., 2019), cytotoxic activity of all the SV_95_-CTLs was determined by CytoTox 96^®^ Non-Radioactive Cytotoxicity Assays. The target cells including T2 cells pulsed with either Mart-1 or SV_95–1_ peptide at 5 μg/ml and prostate cancer cells (LNCap and PC-3) were prepared at a concentration of 4 × 10^5^/ml. The target cells with a number of 2 × 10^4^ were mixed with either TIL2080 cells or the corresponding SV_95_-specific CTLs at ratios of 1:50, 1:25, and 1: 12.5 and a final volume of 100 μl and incubated at 37°C for 3.25 h after spinning down. Ten micro liter of 10× lysis buffer was added into the corresponding wells for maximum release, and incubated at 37°C for additional 45 min. Fifty micro liter of supernatant was taken and transferred into a fresh ELISA plate (MaxiSorp, NUNC, Denmark) after spinning down, and 50 μl of substrate was added into each well and mixed well. The plate was then sealed and incubated at room temperature in the dark for 30 min. Fifty micro liter of stop solution was added into each well, and the plate was read on an ELISA reader at a wavelength of 490 nm. Percent of specific lysis is calculated as follows: % Cytotoxicity = (Experiment – Effector Spontaneous – Target Spontaneous) / (Target Maximum – Target Spontaneous) × 100 (Li et al., 2017; Shao et al., 2017).

### MHC Restriction Assay

Major histocompatibility complex restriction assay was performed using the same method of above cytotoxicity assay except an addition of blocking antibodies including anti-MHC class I antibody (clone BB7.2, BD Pharmingen, San Diego, CA, United States), or anti-MHC II antibody (clone G46-6, Pharmingen, San Diego, CA, United States). Each assay was independently repeated for three times, and each condition was run in triplicates.

### Statistical Analysis

All data is presented as mean ± SEM. Intergroup differences significance was assessed with paired student’s *t* test. The value for statistical significance was established at *p < 0.05, and **p < 0.01. All statistical analyses were performed using GraphPad Prism version 8.4.2 (GraphPad, La Jolla, CA, United States).

## Results

### Selection of Potential SV_95_ CTL Epitopes

CD8^+^ T-lymphocytes are able to recognize peptide antigens presented by specific MHC class I molecules. Based on the immunogenicity and natural sequence of SV_95–1_ peptide formulation, we designed nine novel SV_95_ peptide sequences for further evaluation with the aid of computer-aided algorithm (Table 1). With this modification, both the BIMAS^1^ and SYFPEITHI^2^ algorithm binding scores for SV dramatically improved. Thus, the results from the MHC/peptide binding algorithms suggested several potential CTL epitopes exist within the SV protein. BIMAS still represents the classical epitope prediction program which contains a broad range of human MHC class I motifs, and it was used to predict the half-life of each peptide. BIMAS prediction software allows users to locate and rank the peptides with the length of 8-mer, 9-mer, or 10-mer that contain peptide-binding motifs for MHC class I molecules. For the purposes of confidentiality and patent filing, some amino acids changed in SV_95–6_ and SV_95–7_ peptide sequences in this manuscript were replaced with letter X.

### T2 Cell Binding Analysis of Novel SV_95_ Peptides to HLA-A^∗^02:01

**FIGURE 1:**
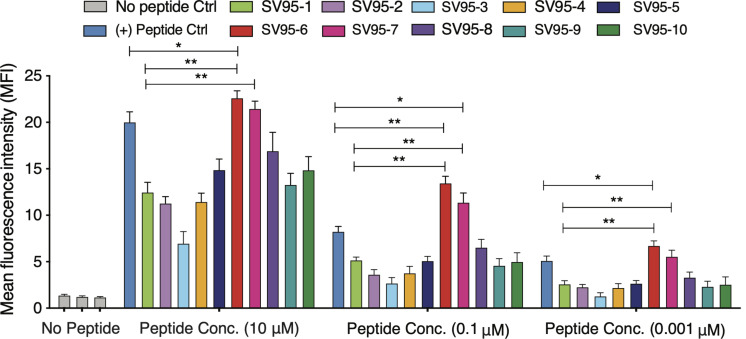
MHC stability assay. T2 cells were incubated with various concentrations of peptide (0.01–10 μM) for 21 h and then stained with anti-MHC I (BB7.2) mAb to quantify the surface expression of HLA-A*0201. POL476 (ILKEPVHGV) was used as a positive control. Either SV_95–6_ peptide or SV_95–7_ peptide significantly binds to MHC I molecules when compared to the control of either SV_95–1_ peptide or positive control peptide (*p < 0.05, **p < 0.01, two-tailed, student’s *t* test, one-way ANOVA).

Ten SV_95_ peptides including a natural SV_95–1_ and nine modified novel SV_95_ peptides listed in Table 1 were tested for binding affinity to HLA-A^∗^0201 by using T2 cells. The results showed that SV_95–6_ peptide has a significant higher binding affinity to T2 cells at the peptide concentrations of 10, 0.1, and 0.01 μM when compared to both the positive control peptide (*p < 0.05, two-tailed, student’s *t* test) and the natural SV_95–1_ peptide (**p < 0.01, two-tailed, student’s *t* test). Similarly, SV_95–7_ peptide has a significant higher binding affinity to T2 cells at the peptide concentrations of both 10 and 0.01 μM when compared to the natural SV_95–1_ peptide (**p < 0.01, two-tailed, student’s *t* test), while SV_95–7_ peptide has a significant higher binding affinity to T2 cells at the peptide concentrations of 0.1 μM when compared to the positive control peptide (*p < 0.01, two-tailed, student’s *t* test) and SV_95–1_ peptide (**p < 0.01, two-tailed, student’s *t* test). As shown in Figure 1, both SV_95–6_ and SV_95–7_ peptides were capable of significantly binding more HLA-A^∗^02:01 molecule on T2 cells among the ten SV_95_ peptides when compared to the positive control peptide as well as SV_95–1_ peptide. These data indicated that SV_95–6_ and SV_95–7_ peptides have a significant binding affinity to HLA-A^∗^02:01 molecule.

### Generation of Artificial APCs

**FIGURE 2:**
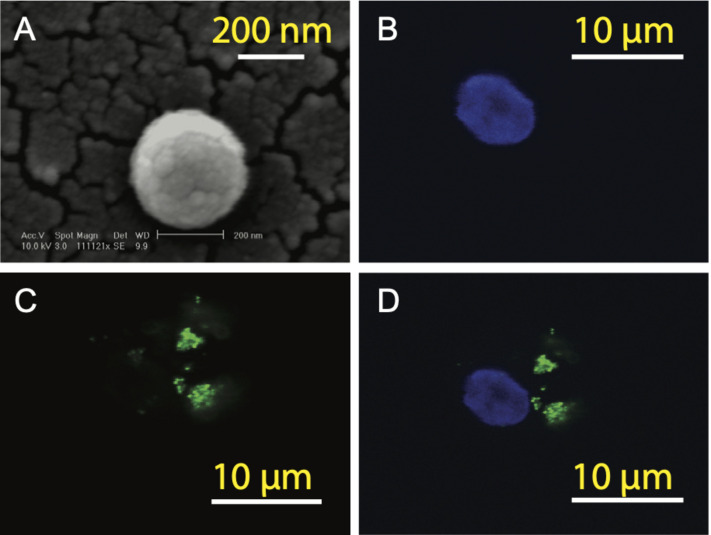
Generation of aAPCs. The aAPCs generated with human DCs and PLGA-NPs encapsulated SV_95_ peptide and coumarin 6 were imaged under a scanning electron microscope (SEM). **(A)** SEM image of the PLGA-NPs with a magnification of 11,112, scale bar = 200 nm. The aAPCs were analyzed using a laser scanning confocal microscope (Leica TCS SP2, Buffalo Grove, IL, United States). **(B)** DAPI channel; **(C)** FITC channels; **(D)** DAPI and FITC channels were overlaid. Scale bar = 10 μm.

In order to prepare aAPCs, PLGA-NPs encapsulated different SV_95_ peptides were first fabricated. All the characteristics of the SV_95_ peptide loaded PLGA-NPs were previously described (Li et al., 2017). In addition, no much difference of the characteristics of PLGA-NPs were found among the PLGA-NPs products fabricated with different SV_95_ peptides (from SV_95–1_ to SV_95–10_). The size of 80% PLGA-NPs displaying diameters was between 150 and 500 nm (Li et al., 2017). A representative image of PLGA-NPs encapsulated SV_95_ peptides was shown in Figure 2A. Second, human imDCs were generated from monocyte cells derived from HLA-A2 positive donors according to our protocol and published methods (Ma et al., 2011; Li et al., 2017). Then, the aAPCs were obtained from the incubation of imDCs with PLGA-NPs encapsulated SV_95_ peptide, followed by a maturation with LPS. Finally, the aAPCs were analyzed under a spectral laser scanning confocal microscope (Leica TCS SP2, Buffalo Grove, IL, United States). DAPI channel showed the nucleus (Figure 2B); FITC channel showed coumarin 6-labeled PLGA-NPs (Figure 2C); overlaid image of DAPI and FITC channels showed the full image of aAPC and demonstrated a colocalization of PLGA-NPs encapsulated SV_95_ peptide (Figure 2D). Scale bar = 10 μm.

### Generation of SV_95_-Specific CTLs

SV95-specific CTLs have been successfully induced according to our protocol and previous publications (Li et al., 2017; Shao et al., 2017). However, the final quantity of CTLs induced may vary from donor to donor. Because the precursor frequency of T cells specific for SV_95_ may not be high enough in some individual donors, therefore, starting number of CD8^+^ T cells could be critical (Supplementary Figure S1).

### SV_95–6_ or SV_95–7_ Peptide Induced CTLs Have a Significant Higher Cytotoxic Activity

**FIGURE 3:**
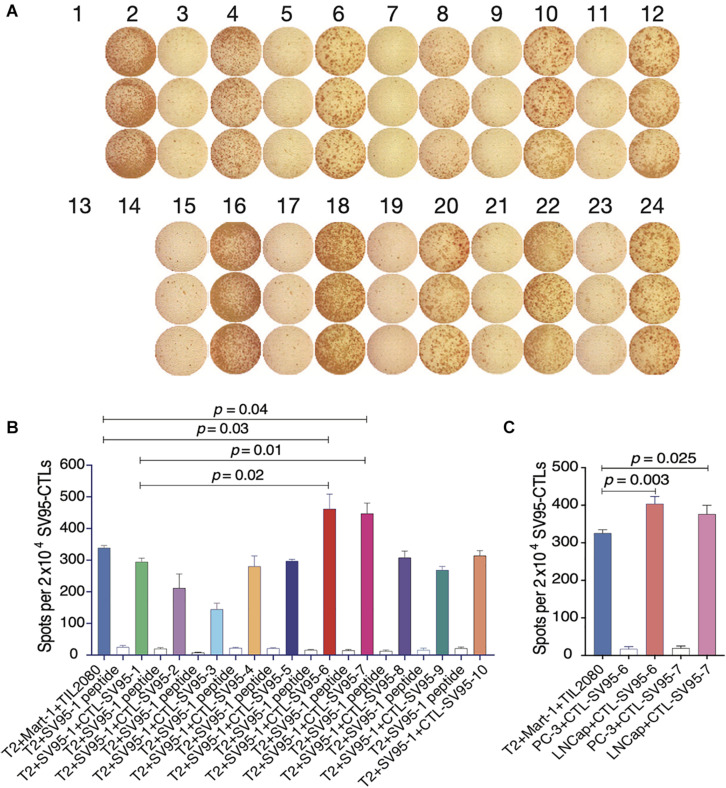
IFN-γ ELISpot assay of the SV_95_-CTL lines. An ELISpot assay was performed for measuring IFN-γ release from the SV_95_ specific CTL lines. **(A)** ELISpot images. Each column represents an experimental group performed in triplicate. **(B)** ELISpot assay results. Either SV_95–6_ or SV_95–7_ specific CTL lines were able to recognize and significantly eliminate SV_95–6_ peptide or SV_95–7_ peptide pulsed T2 cells when compared to either those CTLs generated with the natural SV_95–1_ peptide or TIL2080 cell line (*p* < 0.05, two-tailed, student’s *t* test). **(C)** ELISpot assay results. Both SV_95–6_ and SV_95–7_ specific CTL lines were able to recognize and significantly eliminate prostate cancer (LNCap) cells when compared to the system positive control (TLI2080 cells are able to recognize and eliminate Mart-1 pulsed T2 cells). But, these SV_95–6_ and SV_95–7_ specific CTL lines did not recognize and kill PC-3 cells since it is HLA-A2 negative (*p* < 0.05, two-tailed, student’s *t* test).

Cell-mediated cytotoxicity represents a key mechanism in the immune response to various pathogens and tumors. First, ELISpot assay was performed to assess all the SV_95_-specific CTLs generated with the aAPCs loaded with each corresponding SV_95_ peptide. In this experiment, Mart-1 peptide-pulsed T2 cells incubated with TIL2080 cells were used as the systemic positive control, and T2 cells pulsed with SV_95–1_ peptide were used as the target cells. As shown by the representative ELISpot results in the photomicrograph, IFN-γ was released by those CTL lines and shown as spots (Figure 3A). The spot number generated in each well from T2 target cells was analyzed and graphed in Figure 3B, where data is presented as mean values ± SEM. Each SV_95_-CTL line was tested for three times with different donors. The results showed that the SV_95_-specific CTLs generated with either SV_95–6_ peptide or SV_95–7_ peptide were able to significantly recognize SV_95–1_ peptide pulsed T2 cells when compared to both the CTLs generated with the natural SV_95–1_ peptide (Figure 3B, *p* = 0.01, *p* = 0.02, respectively, two-tailed, student’s *t* test) and TIL2080 cells that recognize Mart-1 peptide pulsed T2 cells (Figure 3B, *p* = 0.04, *p* = 0.03, respectively, two-tailed, student’s *t* test).

Apart from the ELISpot assay from T2 as the target cells, human prostate cancer cell lines including HLA-A2 positive LNCap cells and HLA-A2 negative PC-3 were also included when using both SV_95–6_ specific and SV_95–7_ specific CTL lines to repeat this experiment. The results revealed that both SV_95–6_ and SV_95–7_ specific CTL lines were able to significantly recognize LNCap cells, but not PC-3 cells when compared to the system positive control (Figure 3C, *p* < 0.01, student’s *t* test).

**FIGURE 4:**
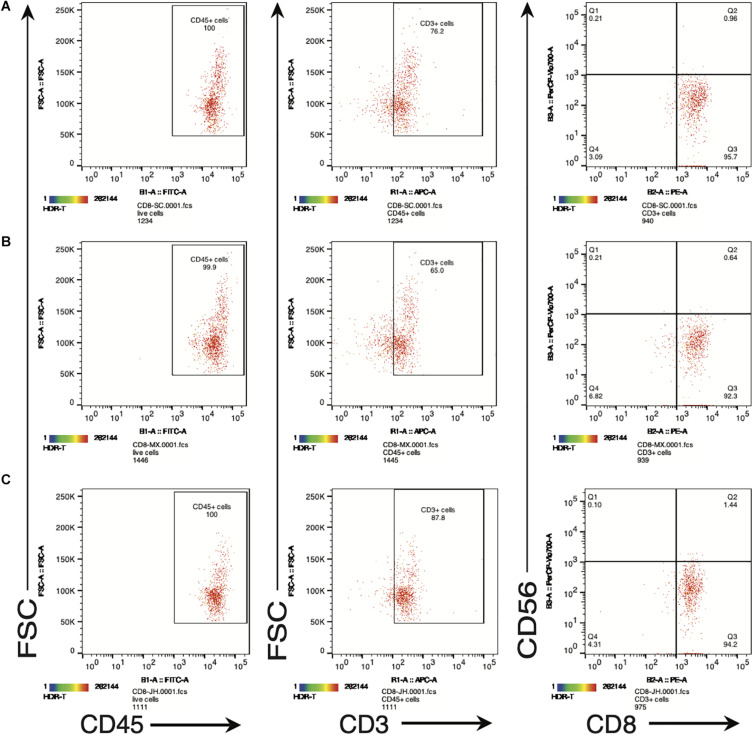
**(A–C)** Are representative FACS plots of SV95-6 specific CTL lines derived from different healthy donors. Phenotype of SV_95–6_ specific CTL line. The SV_95–6_ specific CTL line was stained with human mAbs of CD45, CD3, CD8, and CD56. The flow cytometric results showed that the phenotype of SV_95–6_ specific CTL line is human CD45^+^CD3^+^CD8^+^CD56^–^, so does the SV_95–7_ specific CTL line (FACS plots not shown).

In order to further examine the functional properties of the SV_95_-CTL lines, apart from IFN-γ ELISpot assay, we also checked the biomarkers including human CD45, CD3, CD8, and CD56 on the SV_95_-specific CTL lines by flow cytometry (MACSQuant Analyzer 10, Miltenyi Biotec, Bergisch Gladbach, Germany). The result showed that phenotype of the SV_95_ CTL lines is CD45^+^CD3^+^CD8^+^CD56^–^ (Figure 4). A, B, and C are representative results from different donors.

**FIGURE 5:**
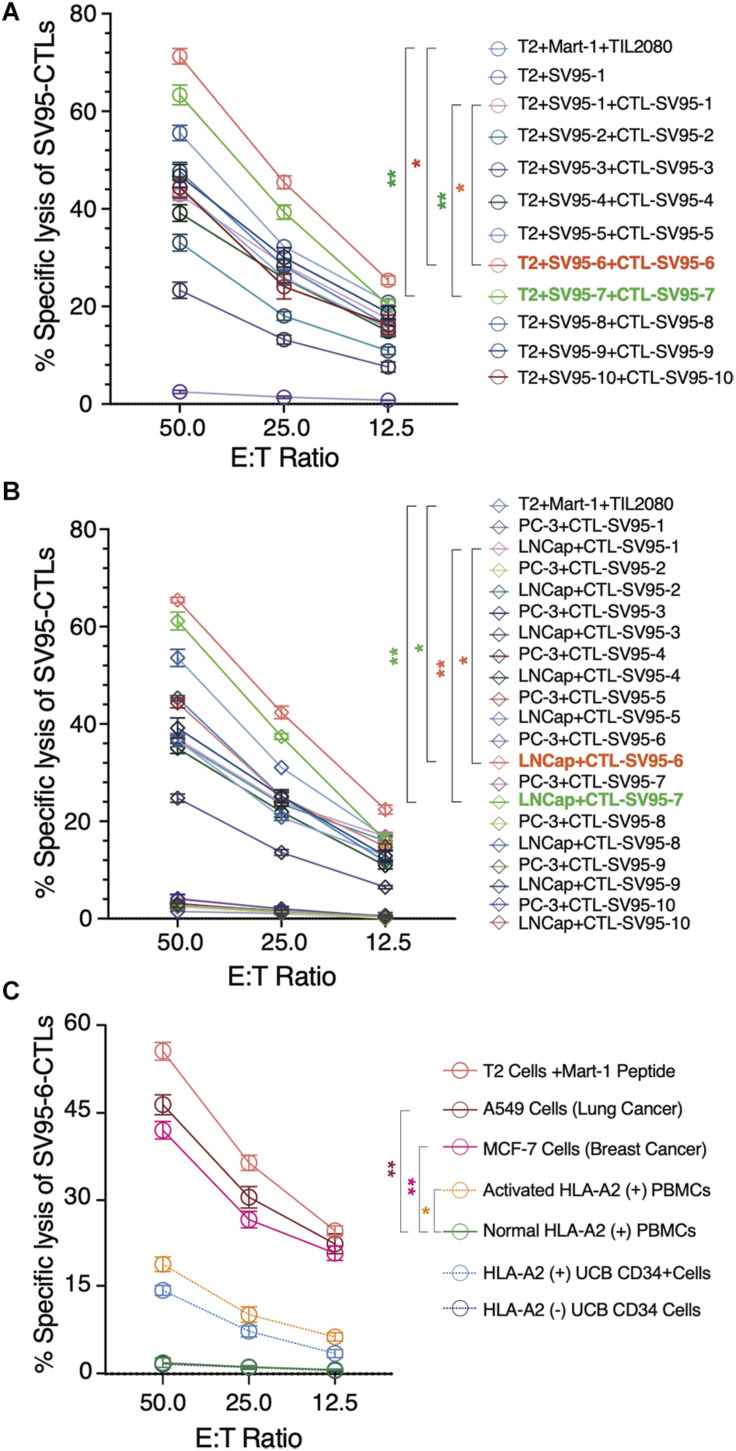
Cytotoxicity assay of the SV_95_-CTL lines. Cytotoxicity assay was performed to test SV_95_ specific CTL lines. SV_95_ peptide pulsed T2 cells were used as the target cells. In addition, both LNCap (HLA-A2 positive cell line) and PC-3 (HLA-A2 negative cell line) were also used as the target cells in this cytotoxicity assay. **(A)** SV_95–1_ peptide pulsed T2 cells were significantly eliminated by both SV_95–6_ and SV_95–7_ specific CTL lines (*p <* 0.05, *p <* 0.01, two-tailed, student’s *t* test). **(B)** Prostate cancer LNCap cells were significantly depleted by both SV_95–6_ and SV_95–7_ specific CTLs (*p <* 0.01, two-tailed, student’s *t* test). **(C)** Lung cancer A549 cells, breast cancer MCF-7 cells were significantly eliminated by SV_95–6_ specific CTL line (*p* < 0.01, two-tailed, student’s *t* test). In addition, activated HLA-A2 (+) PBMCs could also be killed by SV_95–6_ specific CTL line (*p* < 0.05, two tailed, student’s *t* test), but HLA-A2 (+) stem cells were not significantly killed. All the statistics were conducted with one-way ANOVA. *p < 0.05, **p < 0.01.

Along with ELISPot assay, CytoTox 96^®^ Non-Radioactive Cytotoxicity assay was also completed to determine whether these SV_95_-specific CTL lines generated with different SV_95_ peptides (from SV_95–1_ to SV_95–10_) and their corresponding aAPCs could recognize HLA-A2 positive and SV-expressing cancer cells. To do so, T2 cells were used as the target cells after pulsing with SV_95–1_ peptide. The result showed that both SV_95–6_-specific and SV_95–7_-specific CTL lines significantly eliminated the T2 cells pulsed with SV_95–1_ peptide in a dose-dependent manner when compared to both SV_95–1_-specific CTLs (Figure 5A, **p < 0.01, two-tailed, student’s *t* test) and the systemic control TIL2080 cells (Figure 5A, *p < 0.05, **p < 0.01, two-tailed, student’s *t* test).

Aside from evaluating SV_95_-specific CTL lines against target cells of SV_95–1_ peptide pulsed T2 cells, we also assessed the activity of these SV_95_-specific CTL lines against a pair of prostate cancer cell lines including LNCap and PC-3. The results indicated that both SV_95–6_ specific and SV_95–7_ specific CTL lines significantly eliminated LNCap cells in a dose-dependent manner when compared to both SV_95–1_-specific CTLs (Figure 5B, **p < 0.01, two-tailed, student’s *t* test) and the systemic control TIL2080 cells (Figure 5B, *p < 0.05, **p < 0.01, two-tailed, student’s *t* test). However, both SV_95–6_ specific and SV_95–7_ specific CTLs did not eliminate PC-3 cells because PC-3 cell line does not express HLA-A2 molecule. These results suggested that the cytotoxicity of the SV_95_-CTLs might be in an MHC I-restricted manner.

To further assess whether SV_95–6_ specific CTL line or SV_95–7_ specific CTL line is cytotoxic against other cancer types or normal cells, both HLA-A2 and survivin positive cancer cells including lung cancer (A549 cell line) and breast cancer (MCF7) were selected, normal HLA-A2 positive PBMCs together with activated HLA-A2 PBMCs, and normal hematopoietic stem cells (UCB CD34^+^ cells) were also included as the target cells. The results showed that SV_95–6_-specific CTL line significantly eliminated lung cancer cells (A549) and breast cancer cells (MCF7) (Figure 5C. **p < 0.01, two-tailed, student’s *t* test) when compared to normal PBMC control. However, SV_95–6_-specific CTL line is also cytotoxic against activated PBMCs when compared to the target cells of normal PBMCs (Figure 5C. *p = 0.05, two-tailed, student’s *t* test). Although SV_95–6_-specific CTL line recognize normal HLA-A2 positive stem cells (UCB CD34^+^ cells), but this toxicity did not reach significance when compare to normal HLA-A2 negative stem cells (Figure 5C. *p* = 0.085, two-tailed, student’s *t* test). The cytotoxicity data of SV_95–7_-specific CTL line against above cell lines or cells was very similar to that obtained from of SV_95–6_-specific CTL line (data not shown).

### SV_95_-Specific CTLs Recognize and Eliminate Target Cells in MHC Class I-Restricted Fashion

**FIGURE 6:**
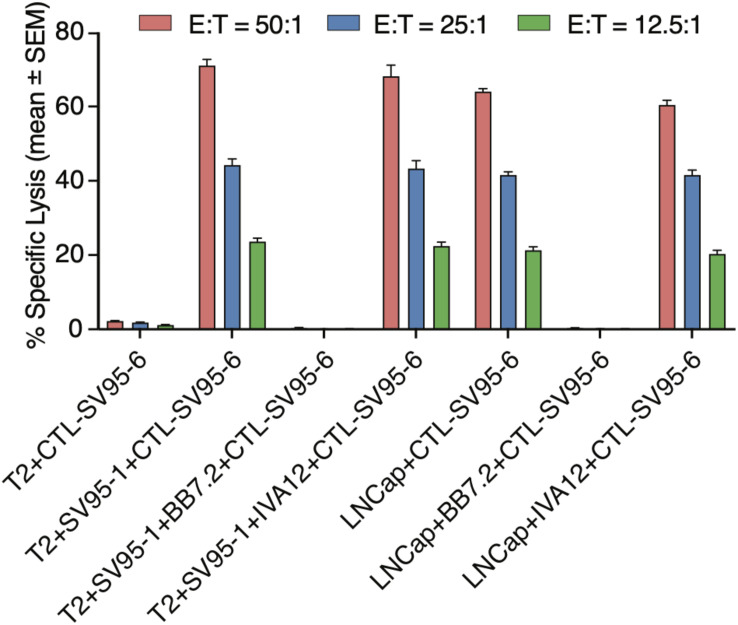
MHC Class I restriction assay. MHC class I restriction assay was performed by a cytotoxic assay of testing SV_95_-specific CTL lines against SV_95–1_ peptide-pulsed T2 cells with the addition of blocking antibody against MHC class I molecule (anti-HLA-A2, BB7.2), or MHC class II molecule (anti-MHC class II, IVA 12). SV_95–6_ specific CTL clone was a representative among the SV_95_ CTL lines. The cytotoxicity of SV_95–6_ specific CTLs was completely blocked by MHC I antibody, but not at all by MHC II antibody.

To demonstrate whether the cytotoxicity of the SV_95_ specific CTLs are in an MHC I-restricted manner, inhibition was performed by adding anti-MHC class I mAb (anti-MHC I, BB7.2), or anti-MHC class II mAb (anti-MHC II, IVA12) in the cytotoxicity assay with the target cells of both SV_95–1_ peptide pulsed T2 cells and LNCap prostate cancer cells. The results showed that cytotoxic activity of the SV_95–6_-specific CTLs was not affected by addition of MHC class II antibody (IVA12), while the cytotoxicity of the SV_95–6_-specific CTLs was thoroughly blocked by addition of MHC class I antibody (anti-HLA-A2, BB7.2). Thus, we demonstrated that the SV_95–6_-specific CTLs generated from HLA-A2 positive healthy donor recognize and eliminate the target cells in an MHC class I fashion (Figure 6).

## Discussion

Survivin is an important TAA that is overexpressed in a variety of cancers but absent or undetectable in normal adult tissues. In addition, expression of SV in cancers correlates with not only inhibition of apoptosis and a decreased rate of cell death, but also resistance to chemotherapy and aggressiveness of cancers (Garg et al., 2016). These characteristics make SV vigorously pursued and an excellent target antigen for cancer immunotherapy. SV peptide epitopes can be recognized by CTLs in the context of MHC I molecules. Besides those *in vitro* studies, presence of spontaneous CTL response against MHC class I restricted SV peptide antigens in patients with lung cancer (Giaccone et al., 2009), breast cancer (Yamanaka et al., 2011), glioma (Liu et al., 2018), and leukemia (Siegel et al., 2004) strongly indicate that CD8^+^ T cell restricted epitopes from SV are of substantial immunotherapeutic value. Similarly, another study demonstrated that SV specific CTL clone isolated and expanded from a cancer patient efficiently lysed a large panel of cancer cells from different origins (i.e., breast cancer, colon cancer, and melanoma cells) (Sorensen et al., 2008). These data support the notion that SV may serve as a universal target antigen for anti-cancer immunotherapy.

T-cell therapies are constantly evolving and improving and providing new options to cancer patients in a variety of cancer types in clinical trials. In this study, we successfully generated SV_95_-specific CTL lines with their corresponding peptides. The CTL lines induced with both SV_95–6_ and SV_95–7_ peptides have significant higher cytotoxicity, and significantly eliminate target cells including T2 cells pulsed with SV_95–1_ peptide and the HLA-A2 positive cancer cells expressing SV when compared to those generated with natural SV_95–1_ peptide and TIL2080 cell line. These results are consistent with a previous study (Sorensen et al., 2008). Our findings not only provide strong evidences that both SV_95–6_ and SV_95–7_ peptides are ready to be presented in the context of HLA-A2 in sufficient amounts to allow recognition by the SV_95_ specific CTL lines, but also provide a compelling rationale for clinical evaluation of SV_95–6_ and SV_95–7_ peptides in clinical trials aimed at eliminating a variety of SV expressing cancers (e.g., lung cancer, breast cancer, prostate cancer, and other cancer types, such as glioma, etc.).

In this study, our strategies were to design and obtain modified peptides with amino acid substitutions at both HLA-A^∗^0201 binding anchor positions to enhance MHC class I binding affinity of the peptides (Fixed Anchor Analogs) in SV_95–2_, SV_95–3_, SV_95–7_, SV_95–9_, SV_95–10_ and at non-anchor positions to enhance the T cell receptor (TCR) binding affinity for the peptide-MHC complex (Heteroclitic Analogs) in SV_95–4_, SV_95–5_, SV_95–6_, SV_95–7_, SV_95–8_, SV_95–9_, and SV_95–10_. During this study, we mainly modified anchor residuals in SV_95–1_ peptide formulation from 2 to 3 amino acids with aromatic hydrophobic residuals such as Tyrosine (A), Tryptophan (W), or aliphatic residual such as Valine (V) at their terminals. Modifications in MHC class I anchor positions were used to improve the binding properties of low-affinity peptides, which can dramatically influence the conformation of the MHC class I peptide groove and thus may have a profound effect on TCR interactions. Although anchor residues can unquestionably impact TCR recognition indirectly by modulating MHC class I binding affinity and selection, but other mechanisms of action proposed that secondary anchor residues can profoundly affect TCR engagement through mechanisms distinct from the alteration of the resting state conformation of the peptide-MHC surface (Miley et al., 2004). Two amino acid side chains of the peptide, the second and the ninth fit into pockets in the MHC I molecule, tightly anchoring MHC I molecule to form a peptide-MHC I complex. The strong interactions of the anchor residuals side chains with their corresponding pockets may permit the effective binding of 10-mer SV_95_ peptides to MHC I molecules. Consequently, we presented the identification of novel antigenic peptide epitopes within the SV_95_ molecule. Most of researchers agree with the importance of the F-pocket region for peptide exchange. Because the binding groove in MHC I molecules is closed at both ends by conserved tyrosine (Tyr) residues, this lead to a size restriction of the bound peptides to usually 8–10 residues with its C-terminal end docking into the F-pocket (Thomas and Tampe, 2017; Wieczorek et al., 2017).

A few publications on novel SV-derived CTL epitopes were found as studies on novel SV peptides are rare. In order to increase the minimal CD8^+^ T cell epitope binding affinity to the HLA-A2.1 allele and subsequently increase the immune responses, Bernatchez et al. (2011) reported that a modification of replacing M with T at position 3 in SV_95–104_ (ELMLGEFLKL) peptide had enhanced HLA-A^∗^0201 binding and induced a stronger T-cell response over its wild type counterpart peptide (ELTLGEFLKL) in selected HLA-A^∗^0201 positive donors. In addition, Xiang et al. (2018) modified SV peptide formulations of SV_95–104_ and SV_96–104_ by substituting the amino acid Threonine (T) to Methionine (M) at the position 97 (ELMLGEFLKL, LMLGEFLKL, herein named SV_95–104_ variant, SV_96–104_ variant) as agonists for use with their polystyrene nanoparticle (PSNP) vaccines. Their results showed that CD8^+^ T cell epitope of SV_96–104_ variants (LMLGEFLKL and AAYLMLGEFLKL) were able to induce the HLA-A2.1 restricted CD8^+^ T cell responses to SV_95–104_ variant (ELMLGEFLKL) upon immunization with SV_96–104_ variant-PSNP or AAYLMLGEFLKL-PSNP vaccine formulations. In our study, to find stronger anchors and introduce higher stability to MHC class I molecules, we modified both N-terminus and C-terminus in some of the SV_95–104_ peptide.

The algorithms used in this study were proved to be overall useful predictors of immunogenic SV peptides for HLA-A2 type. These HLA-A2-restricted SV peptides fulfill desired criteria for immunogenicity. To test the discrepancy between predicted affinity, actual avidity, and immunogenic function of the peptide sequences, we measured the peptides binding capacity to HLA-A2 molecules. To further evaluate the effectiveness of these novel SV_95_ peptides, we generated the SV_95_-induced specific CTLs by using CD8^+^ T cells derived from HLA-A2 positive donors and aAPCs that were made from human DCs and the corresponding SV_95_ peptides loaded PLGA-NPs, as opposed to SV peptide pulsed human DCs as the APCs utilized by other scientists (Schmitz et al., 2000; Khalaf et al., 2019). Because we were able to demonstrate that aAPCs are superior to DCs at inducing CTL responses (Li et al., 2017), we believe that the SV_95_-CTLs we generated benefit from our SV_95_ peptide loaded aAPCs.

It is known that MHC I molecules become unstable in the absence of binding peptides. In order to provide a stable supply of peptides, we used PLGA-NPs-based aAPCs to continuously present SV_95_ peptide to (naïve) CD8^+^ T cells. High structural integrity of PLGA-NPs enhances the stability, which then increases the drug loading and prolongs the drug release time. This phenomenon may significantly contribute to the success of SV_95_-specific CTL generation. In addition, adequately designed and engineered polymeric particles can contribute to a desirable drug delivery characterized by sustained drug release, prolonged drug action, reduction in the therapeutic dose, and improved patient compliance.

It should be noted that SV_95_-specific CTL lines generated by novel SV_95–6_ and SV_95–7_ peptides have been examined only on the target cells of T2 cells and other limited cancer cell lines including lung cancer, breast cancer, and prostate cancer. Other common cancer cell lines such glioma and melanoma were not included. In this study, we mainly concentrated on demonstrating the feasibility of novel SV_95_ peptides when inducing CTL lines and testing if the new CTL lines generated with novel SV_95–6_ or SV_95–7_ peptide are superior or significant stronger than those induced with original SV_95–1_ peptide. In addition, one more thing we want to point out based on this study, the SV_95_-specific CTLs are slightly cytotoxic to both activated HLA-A2 positive PBMCs (but not cytotoxic to normal PBMC) and hematopoietic stem cells (Figure 5C). This phenomenon has been previously reported (Leisegang et al., 2010), because the most widely expressed tumor antigen targets for malignant cells are often also expressed on non-malignant cells (Scherer et al., 2019). However, this fratricide phenomenon will not affect the space for CTLs in clinical applications but needs to be noted.

## Conclusion

We presented the identification of nine novel SV_95_ peptide sequences restricted to HLA-A2 molecule, and found SV_95–6_ and SV_95–7_ peptides are new HLA-A2-restricted CTL epitopes that are able to successfully generate SV_95–6_- or SV_95–7_-specific CTL line with a significant higher cytotoxicity compared to SV_95–1_-specific CTL line. These SV_95–6_- and SV_95–7_-specific CTLs not only recognize the cancer cells, which express SV protein, but also efficiently eliminate these cancer cells. We demonstrated that SV_95–6_ and SV_95–7_ peptides could induce CTL response in the context of HLA-A2 molecules. This study implies that two novel SV_95_ peptides with much stronger immunogenicity that could justify their application in the clinical immunotherapy on the cancer patients.

## Data Availability Statement

The raw data supporting the conclusions of this article will be made available by the authors, without undue reservation.

## Ethics Statement

The studies involving human participants were reviewed and approved by Institutional Review Board at University of California San Diego. The patients/participants provided their written informed consent to participate in this study.

## Author Contributions

QC, GJ, XZ, YB, YZ, and WM carried out and participated in all of the experiments including T2 peptide binding assay, preparation and characterization of PLGA-NPs, PBMC isolation, DC culture and preparation of aAPCs, CTL generation, ELISpot assay, cytotoxicity assay, data process, and manuscript preparation. CO supervised studies related to characterization of PLGA-NPs, DC loading, and imaging analysis. BM and WM designed, supervised, and coordinated the entire study, performed the statistical analysis, and drafted the manuscript. All authors read and approved the final manuscript.

## Conflict of Interest

The authors declare that the research was conducted in the absence of any commercial or financial relationships that could be construed as a potential conflict of interest.

## Footnotes

1 http://tools.iedb.org/immunogenicity/

2 http://www.syfpeithi.de/

## Supplementary Material

FIGURE S1 Growth curves of SV_95_-specific CTL lines. PBMC cell number, initial CD8^+^ T cell number and the final cell number of each SV_95_-specific CTL line generated from each healthy donor were graphed in this figure. Legends in this figure are donor sample IDs.
