# Supplementary figures and images for: Novel Survivin Peptides Screened With Computer Algorithm Induce Cytotoxic T Lymphocytes With Higher Cytotoxic Efficiency to Cancer Cells
Source: Front Mol Biosci. 2020 Sep 2;7:570003. doi: 10.3389/fmolb.2020.570003 (PMC7496070; doi:10.3389/fmolb.2020.570003)

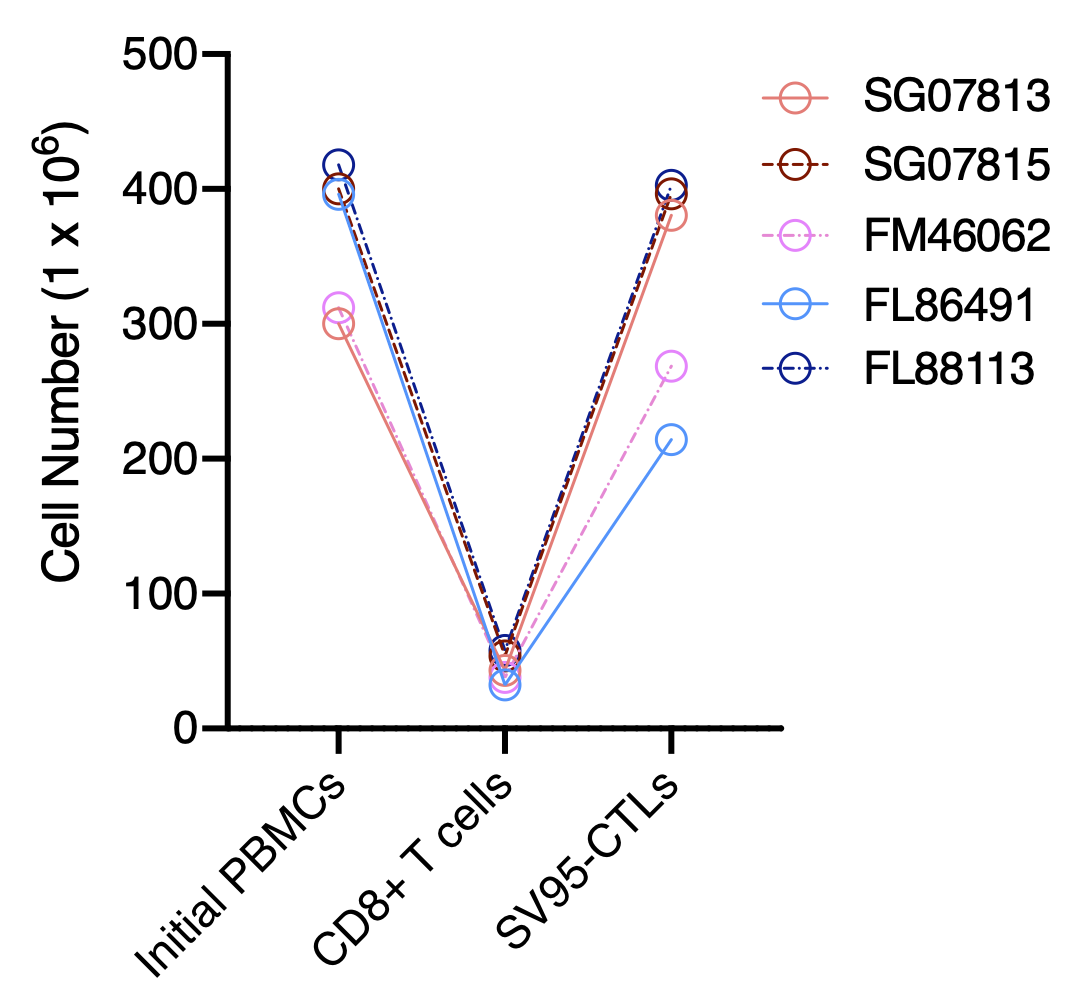

Supplement: FIGURE S1 — Growth curves of SV95-specific CTL lines. PBMC cell number, initial CD8+ T cell number and the final cell number of each SV95-specific CTL line generated from each healthy donor were graphed in this figure. Legends in this figure are donor sample IDs. [file Image_1.tiff]
